# WAWA: Wavelet Analysis-Based Watermarking Authentication for GNSS Civil Signal with Immediate Symbol-Level Verification

**DOI:** 10.3390/s25216615

**Published:** 2025-10-28

**Authors:** Xinyu Tang, Xiaomei Tang, Honglei Lin, Yi Wu, Guangfu Sun

**Affiliations:** 1 College of Electronic Science and Technology, National University of Defense Technology, Changsha 410073, China; tangxinyu19@nudt.edu.cn (X.T.); tangxiaomei@nudt.edu.cn (X.T.); linhonglei@nudt.edu.cn (H.L.); wuyi@nudt.edu.cn (Y.W.); 2 Key Laboratory of Satellite Navigation Technology, National University of Defense Technology, Changsha 410073, China

**Keywords:** GNSS, signal authentication, wavelet analysis, digital watermarking, symbol-level authentication, public key cryptography, anti-spoofing, WAWA

## Abstract

Existing GNSS authentication schemes suffer from critical drawbacks such as high verification latency and prohibitive memory requirements, leaving time-sensitive applications vulnerable to spoofing. The core challenge is the inability to transmit strong, real-time cryptographic credentials through the bandwidth-limited GNSS signal. This paper introduces WAWA, a Wavelet Analysis-based Watermarking Authentication scheme that operates at the physical layer of the GNSS signal. The central innovation of WAWA is its use of the wavelet domain to achieve a high-capacity data channel, allowing a complete public-key digital signature to be embedded directly within the signal structure. This enables receivers to perform immediate, symbol-level authentication using a public key, which fundamentally removes the verification delay and reliance on time synchronization seen in conventional methods. Furthermore, it eliminates the need for large memory buffers, a critical barrier for resource-constrained devices. We present the complete design of the watermark generation, embedding, and extraction process, alongside a novel dual-path verification framework adaptable to both standalone and network-assisted receivers. Performance analysis shows that WAWA achieves immediate authentication while offering superior effective bandwidth and maintaining low memory overhead. Although it introduces a controllable signal correlation loss, validated through both theoretical modeling and simulation, WAWA presents an exceptional balance of security, immediacy, and resource efficiency, offering a promising new paradigm for ensuring trustworthy PNT sensor data in time-critical and resource-sensitive applications, particularly in large-scale sensor networks and autonomous systems.

## 1. Introduction

**Figure 1:**
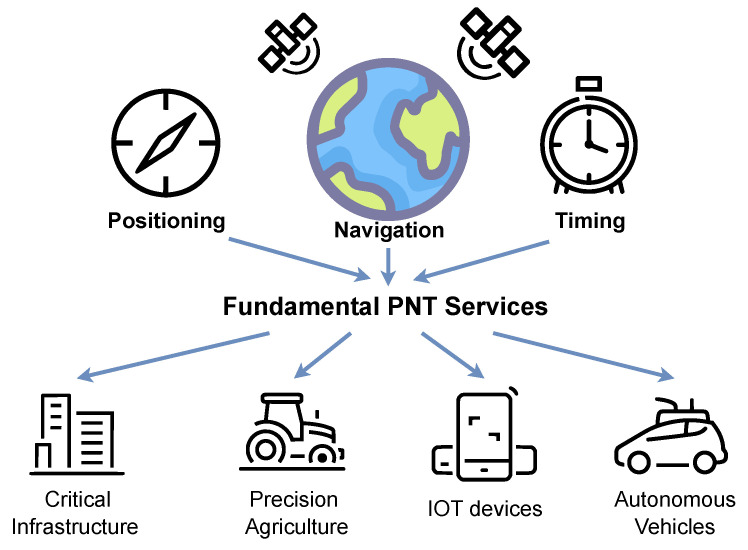
The fundamental Positioning, Navigation, and Timing (PNT) services provided by GNSS and their key applications in modern society.

Global Navigation Satellite Systems (GNSS) have evolved into a ubiquitous and indispensable global utility. They provide the foundational Positioning, Navigation, and Timing (PNT) data that underpin a vast array of modern technologies, from synchronizing critical infrastructure like power grids and financial networks to enabling precision agriculture and facilitating emerging technologies such as the Internet of Things (IoT) and autonomous vehicles [1]. The integrity of PNT information is therefore of paramount importance. Modern society’s dependence on GNSS extends beyond simple navigation, encompassing Remote Sensing reflectometry for Earth observation and climate research, intelligent transportation systems in smart cities, and multi-sensor integration for autonomous operations [2,3]. This widespread reliance makes the reliability and security of GNSS services increasingly critical, as illustrated in Figure 1.

**Figure 2:**
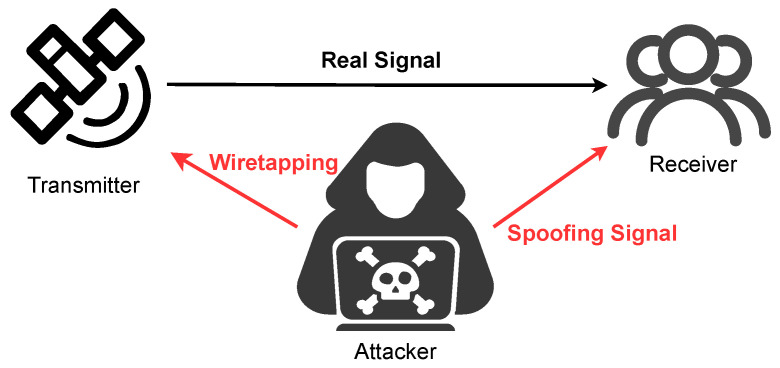
Asimplified illustration of a GNSS spoofing attack, where a malicious attacker generates spoofing signals to mislead a target receiver.

However, the open and unencrypted nature of civilian GNSS signals makes them fundamentally vulnerable to manipulation [4,5]. While threats include relatively simple signal replay attacks (meaconing), the most pernicious form is generative spoofing, where an adversary broadcasts entirely counterfeit signals. By doing so, a spoofer can gain arbitrary control over a receiver’s calculated position and time, making it a severe threat to systems that depend on reliable PNT services [6,7]. The development of robust defenses against such generative attacks is therefore of paramount importance and the primary focus of this paper. These attacks have already demonstrated the potential for significant economic disruption and catastrophic failures (a simplified illustration is provided in Figure 2).

Recent analysis of GNSS interference incidents reveals substantial economic consequences, with a single spoofing event estimated to cost GBP 1.28 million in the UK alone due to transportation delays, flight disruptions, and reduced operational efficiency [8]. The sophistication of spoofing technology has increased dramatically, with documented cases of aviation incidents where aircraft lost all navigation systems and drifted 80 miles off course, requiring emergency navigation procedures to complete flights safely [4]. Critical infrastructure sectors, including maritime shipping, precision agriculture, and financial timing systems, face escalating risks as low-cost spoofing devices become increasingly accessible, with the potential to disrupt supply chains, compromise automated farming operations, and destabilize time-sensitive financial transactions [9]. Consequently, developing robust methods to authenticate GNSS signals and guarantee their trustworthiness has become a critical area of research.

Current approaches to combat GNSS spoofing can be broadly divided into two categories. The first category involves non-cryptographic methods, such as monitoring signal characteristics at the physical layer [10,11] or performing consistency checks at the receiver level [4,9,12]. While these techniques can detect certain anomalies, they often lack the mathematical certainty of cryptographic methods and can be circumvented by more sophisticated attacks or fail in complex signal environments [13]. This paper focuses on the second category: cryptographic signal authentication, where security data is embedded within the signal itself to cryptographically verify its origin and integrity [14]. The two most prominent frameworks in this domain are Navigation Message Authentication (NMA) and Spreading Code Authentication (SCA).

NMA schemes operate by embedding authentication data into the navigation message broadcast by the satellites. The leading example is the Galileo Open Service Navigation Message Authentication (OSNMA) [15], which is based on the Timed Efficient Stream Loss-tolerant Authentication (TESLA) protocol [16]. TESLA ingeniously uses delayed key disclosure over a public channel to achieve authentication with computationally efficient symmetric cryptography. However, this mechanism comes with a significant operational drawback: receivers must buffer incoming data and wait for a period of seconds to tens of seconds for the corresponding key to be broadcast, creating a substantial verification latency [17]. Moreover, TESLA’s security model depends on a loose time synchronization between the satellite and the receiver. This dependency has been identified as a potential vulnerability, as attackers could manipulate a receiver’s sense of time to bypass the authentication check [18,19,20].

SCA schemes aim to protect the signal’s pseudorange measurements by authenticating the spreading code itself [21]. A well-known example is the Chimera protocol proposed for GPS [22], which embeds encrypted markers known as Secure Pilot Components (SPCs) into the signal. In practice, SCA is often used in conjunction with NMA to provide comprehensive protection. However, this hybrid approach inherits a critical flaw from NMA. The keys required to verify the SCA markers are typically transmitted via the slow NMA layer. This forces the receiver to buffer large quantities of raw baseband signal data until the NMA key is finally received and processed. The resulting memory requirement can reach several megabytes [23,24], a demand that is often prohibitive for the low-cost, power-constrained hardware typical of large-scale IoT deployments and other resource-sensitive applications.

At its core, the fundamental challenge facing existing cryptographic GNSS authentication is the efficient real-time delivery of strong cryptographic credentials. While the architectural elements for this, such as a Public Key Infrastructure (PKI), are increasingly mature—with Galileo deploying its own and schemes like TrustCNAV [25] offering advanced designs—the physical means of transmission remain a critical bottleneck. The existing satellite-to-earth channel simply lacks the bandwidth to carry a full public-key digital signature without incurring the unacceptable delays of NMA or the high memory costs of SCA. Therefore, this paper focuses on solving this specific transmission problem.

To overcome this fundamental challenge, this paper proposes a Wavelet Analysis-based Watermarking Authentication (WAWA) scheme. WAWA is designed to create a high-capacity side channel by embedding authentication data directly into the physical layer of the GNSS signal using a wavelet-domain digital watermark. This enables the transmission of a full public-key digital signature, allowing for immediate verification at the receiver. The following main contributions of this work are threefold:

1. Novel Authentication Architecture for Immediate Verification: We introduce the WAWA scheme, which integrates public-key cryptography with wavelet-domain watermarking. This architecture facilitates the transmission of a complete digital signature within the physical signal layer, thereby eliminating the verification delay and time-synchronization dependency that are inherent weaknesses of TESLA-based protocols.
2. Low-Memory, Dual-Mode Verification Framework: We design a flexible, dual-path verification framework adaptable to diverse receiver capabilities. It features a “slow path” for fully standalone receivers and a “fast path” for network-assisted receivers, enabling the architecture to support a wide range of resource-constrained devices without the need for large data buffers.
3. Comprehensive Performance Modeling and Simulation Verification: We develop theoretical models to evaluate the scheme’s detection performance and its impact on the primary GNSS signal and validate the models through simulation. This analysis quantifies the trade-offs between key system parameters, providing a clear framework for system design and optimization.

The remainder of this paper is structured as follows: Section 2 details the system model and the proposed WAWA scheme. Section 3 presents the results of the theoretical performance analysis and an impact assessment on legacy receivers. Section 4 discusses the interpretation and implications of these results in comparison to existing schemes. Finally, Section 5 concludes the paper.

## 2. Materials and Methods

### 2.1. Foundations of the WAWA Scheme

To thoroughly understand the WAWA scheme, this section establishes its theoretical and technical foundations, including the signal model, cryptographic framework, and the principles of wavelet-domain embedding.

#### 2.1.1. GNSS Baseband Signal Model

**Figure 3:**
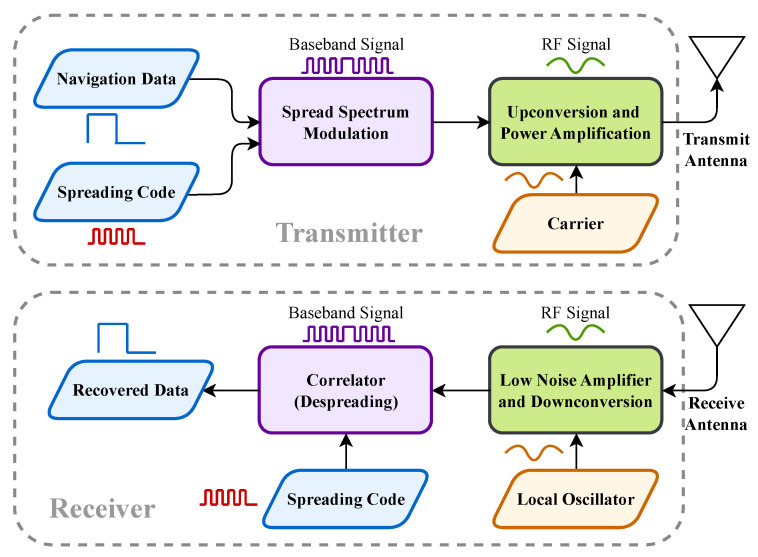
A simplified framework for traditional GNSS Direct Sequence Spread Spectrum (DSSS) signal modulation and demodulation. The waveforms are illustrative, representing the digital nature of the baseband signal and its modulated form at the RF stage.

Modern civil GNSS signals, such as those from GPS and Galileo, are built upon Direct Sequence Spread Spectrum (DSSS) technology. Figure 3 provides a simplified illustration of a typical DSSS modulation and demodulation process. At the transmitter (satellite), low-rate navigation data bits are modulated by a high-rate pseudorandom noise (PRN) spreading code, which spreads the signal’s energy across a wide bandwidth. This baseband signal is then modulated onto a carrier frequency and broadcast towards Earth.

At the receiver, the incoming signal is down-converted and processed to remove carrier frequency and Doppler shifts, yielding the baseband signal. A key observation is that the structure of this baseband signal within a single navigation symbol period, $T_{sym}$, is stable and predictable. The WAWA scheme leverages this stability by embedding authentication information directly at the baseband signal level, treating the inherent signal structure as a carrier for the watermark. For the remainder of this paper, the term “Original GNSS Signal”, denoted as s(t), refers to this baseband signal within one symbol period.

#### 2.1.2. Cryptographic Cornerstone: Public Key Cryptography for Immediate Authentication

To fundamentally eliminate the verification delays and time-synchronization risks associated with the TESLA protocol, the WAWA scheme is built upon Public Key Cryptography (PKC). Unlike symmetric cryptography, which requires the sender and receiver to share a secret key, PKC employs a mathematically linked key pair for each user: a private key ($K_{priv}$) and a public key ($K_{pub}$). The private key is held securely by the originator (e.g., the GNSS control segment), while the public key can be distributed openly to all receivers.

Digital signatures are a primary application of PKC and are central to WAWA. To generate a signature, the sender uses their private key $K_{priv}$ to sign a message *M* (e.g., a portion of the navigation data). This produces a unique cryptographic value, the digital signature Sig, which is intrinsically tied to both the message and the sender’s identity. The message and signature are then transmitted to the receiver. The receiver uses the sender’s public key $K_{pub}$ to perform a verification algorithm on the received message and signature. A successful verification provides strong cryptographic assurance that the message originated from the legitimate sender and that its content has not been altered in transit.

**Figure 4:**
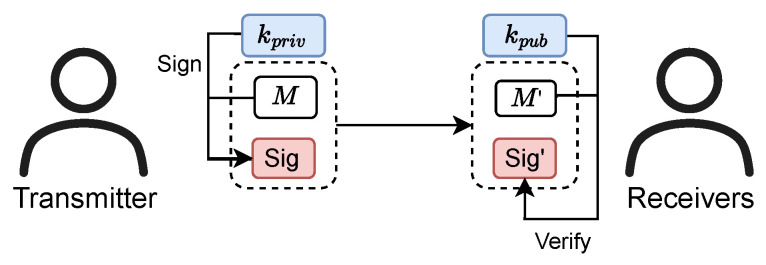
The fundamental workflow of a PKC digital signature scheme.

The critical advantage of digital signatures for GNSS authentication is that verification depends only on the public key and the received data. This means verification can occur the moment a complete message and signature have been received, without waiting for any subsequent key disclosures. This property is the foundation for WAWA’s real-time verification capability. The public-key framework outlined in TrustCNAV [25] provides a relevant model for the cryptographic operations within WAWA.The fundamental workflow of this process is depicted in Figure 4.

#### 2.1.3. Embedding Strategy: Digital Watermarking

To transmit the relatively long digital signature over the GNSS channel, WAWA employs digital watermarking. Digital watermarking is a technique of information hiding that embeds auxiliary data into a host digital medium [26]. A key requirement is that the embedding process should have a minimal and controllable impact on the host medium’s primary function and be robust against routine processing or noise. This makes it an ideal technique for embedding authentication data within the noise-limited GNSS broadcast signal.

A powerful watermarking strategy is transform-domain embedding. In this approach, the host signal is first mapped into a different domain using a mathematical transform. The watermark is then embedded by modifying the coefficients in this new domain. Finally, an inverse transform returns the signal to its original domain. By choosing an appropriate transform, it is possible to leverage its properties to achieve higher embedding capacity, better imperceptibility, or stronger robustness than direct time-domain methods.

#### 2.1.4. Embedding Domain: Discrete Wavelet Transform (DWT)

The WAWA scheme is founded upon the orthogonal Discrete Wavelet Transform (DWT), a signal processing tool uniquely suited for analyzing non-stationary signals. This capability is paramount for GNSS applications, where receiver dynamics can induce time-varying frequency characteristics. Unlike the Fourier transform’s global frequency representation, DWT provides a superior time-frequency analysis by decomposing a signal into components localized in both domains,

(1) $$\begin{array}{c} s\overset{\mathrm{DWT}}{\to }\left\{A_{l},D_{l},D_{l-1},\ldots ,D_{1}\right\} \end{array}$$

where $A_{l}$ represents the low-frequency approximation coefficients and $\left\{D_{j}\right\}$ are the detail coefficients across multiple higher-frequency levels. Crucially, unlike the Fourier transform, which uses fixed sine and cosine functions as its basis for all signals, a wavelet transform utilizes a family of basis functions (wavelets) that are localized in both time and frequency. This adaptability allows DWT to more efficiently capture transient or non-stationary features within a signal.

**Figure 5:**
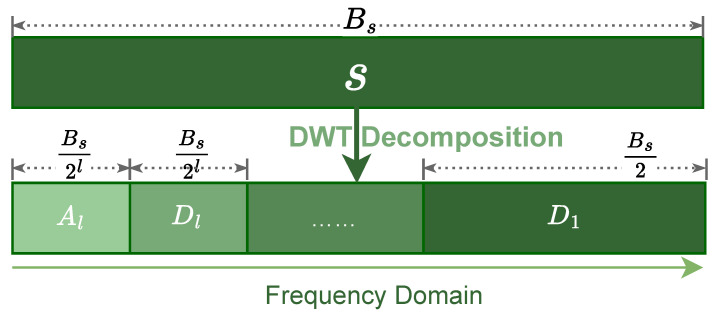
Conceptual representation of DWT as a multi-resolution analysis in the frequency domain. An l-level decomposition partitions the original signal’s spectrum into one low-frequency approximation sub-band ($A_{l}$) and l high-frequency detail sub-bands ($D_{1}$ to $D_{l}$), each with a specific bandwidth.

Conceptually, this decomposition is equivalent to a multi-resolution filter bank that partitions the original signal’s spectrum into a series of orthogonal, dyadic sub-bands, as depicted in Figure 5. This illustrates the fundamental principle of creating distinct spectral partitions for analysis and modification.

**Figure 6:**
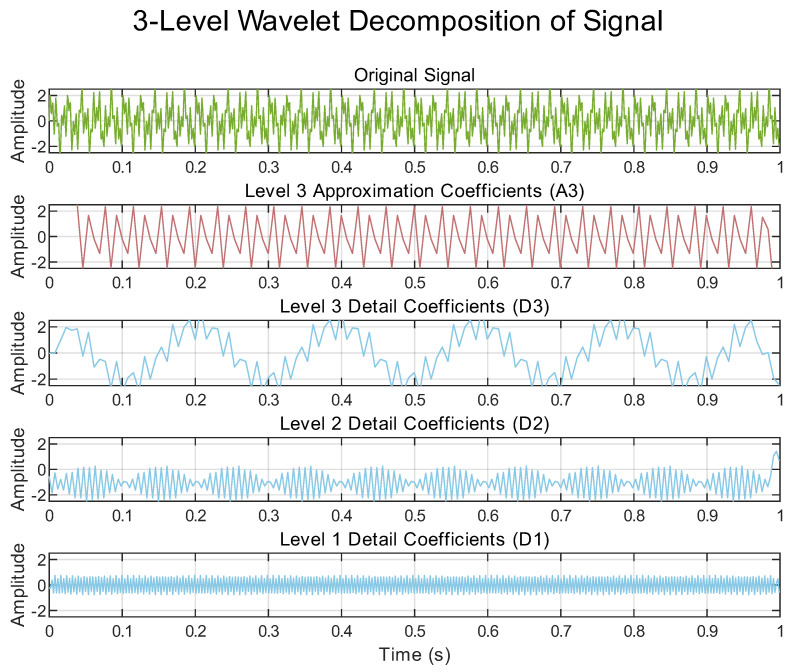
An illustrative time-domain view of a 3-level DWT decomposition on a generic signal. This figure demonstrates the key property leveraged by WAWA: the original signal’s temporal structure is preserved within each decomposed sub-band (A3, D1–D3), enabling a time-series watermark to be embedded in a specific frequency layer.

The true power of DWT for WAWA’s embedding strategy, however, is revealed by observing this decomposition in the time domain, as illustrated in the concrete example of Figure 6. While Figure 5 shows the what (the spectral division), Figure 6 demonstrates the critical how: Each set of sub-band coefficients is itself a time series that preserves the temporal features of the original signal corresponding to its respective frequency band. It is this dual localization in both time and frequency that makes DWT the ideal transform for our application. It allows us to treat a specific detail sub-band as an independent, lower-rate ’channel’ into which we can add our time-structured watermark signal, w(t), with minimal interference to other components. This principle is central to the embedding process detailed in Section 2.2.2.

WAWA’s embedding strategy is designed to directly exploit this multi-resolution framework. Rather than superimposing the watermark across the entire time-domain signal, WAWA performs a targeted insertion into one or more of the detail coefficient sub-bands. This approach affords the system designer a crucial degree of freedom: the ability to place the watermark in spectral regions of lower host signal energy, thereby maximizing watermark robustness while minimizing interference with the primary navigation components.

### 2.2. Design of the WAWA Scheme

This section details the end-to-end design of the WAWA scheme, from watermark generation at the transmitter to the dual-path verification at the receiver.

#### 2.2.1. System Architecture

**Figure 7:**
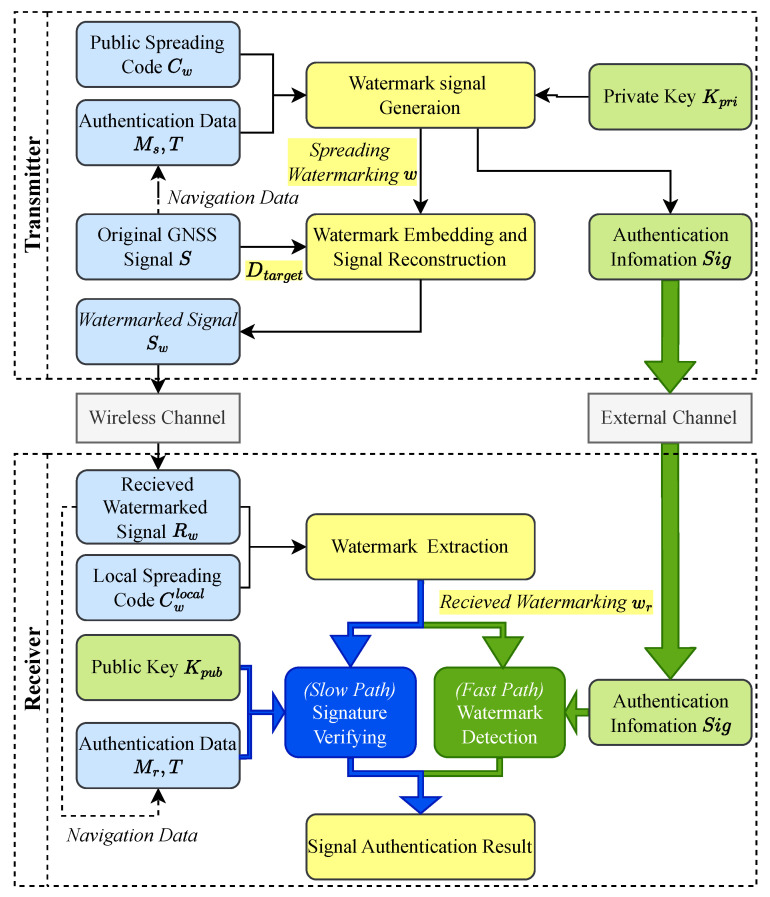
System architecture of the WAWA scheme. The conceptual modules are color-coded: blue for navigation signal processing, green for cryptographic operations (using private key $K_{priv}$ and public key $K_{pub}$), and yellow for watermark processing. The flow of data and processing steps from navigation message generation to the final authenticated output is illustrated.

The overall architecture of the WAWA scheme is depicted in Figure 7. The system is composed of cryptographic, watermark processing, and navigation signal processing modules, logically separated into a transmitter-side process and a receiver-side process.

At the transmitter, e.g., the GNSS ground control segment, the process begins with the cryptographic module, which generates a digital signature Sig for a block of navigation data using a private key $K_{priv}$. This signature, which serves as the core authentication payload, is then transformed into a robust, spread-spectrum watermark signal *w* by the Watermark Generation module. The central innovation resides in the Watermark Embedding module. Instead of directly modifying the time-domain signal, it first decomposes the original GNSS baseband signal *s* into its constituent time-frequency components via the DWT. The watermark *w* is then embedded by modifying a specific set of these components. Finally, an Inverse DWT IDWT reconstructs the signal, yielding the final watermarked signal $s_{w}$, ready for uplink and broadcast.

At the receiver, the process is designed for both efficiency and flexibility. After the front-end has acquired and tracked the signal, the Watermark Extraction module performs a DWT on the received baseband signal $r_{w}$ to isolate the sub-bands containing the watermark. It then despreads these components to obtain the raw extracted watermark $w_{r}$. At this point, WAWA’s key architectural feature comes into play: a dual-path verification framework.

- The slow path is designed for fully autonomous, standalone receivers. It performs a hard-decision decoding on the extracted watermark $w_{r}$ to reconstruct the full digital signature, which is then verified using the public key $K_{pub}$. This path guarantees authentication without reliance on any external communication.
- The fast path is designed for network-assisted receivers that prioritize speed. In this mode, the receiver obtains the authentic signature Sig in advance from a trusted, high-speed channel, e.g., a cellular network. It then uses this authentic signature as a local template for a rapid correlation detection against the soft-decision values of the extracted watermark $w_{r}$, enabling near real-time authentication.

Both paths culminate in a definitive signal authentication result, providing a versatile solution adaptable to a wide range of application requirements and device capabilities.

#### 2.2.2. Wavelet Domain Embedding Process

**Figure 8:**
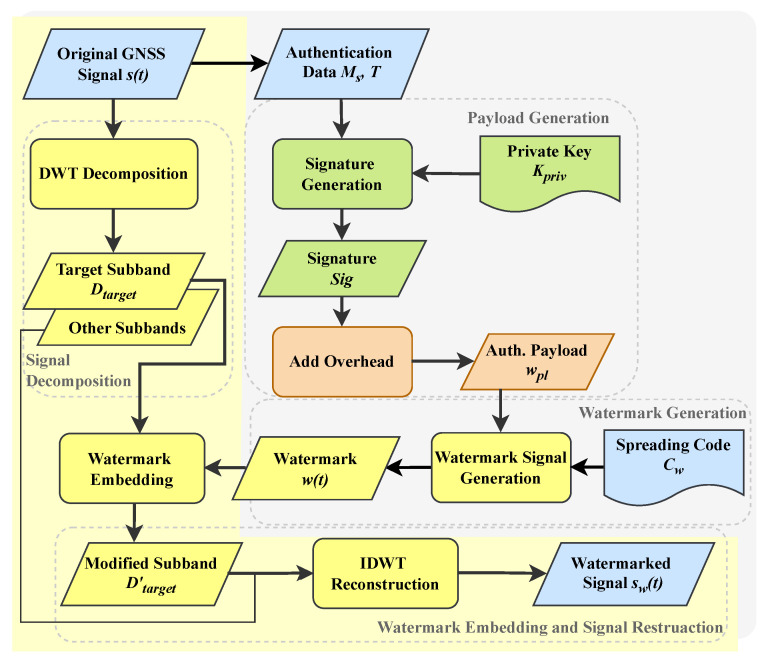
Transmitter flowchart showing watermark generation and embedding. The process takes the original signal s(t) and authentication data (M,T) to produce a watermarked signal $s_{w}\left(t\right)$ using a private key ($K_{priv}$) and a spreading code ($C_{w}$).

WAWA utilizes the time-frequency localization property of DWT to embed the watermark with precise energy control, minimizing its impact on critical signal components. The core embedding mechanism is illustrated in the yellow-highlighted portion of Figure 8.

The process begins by applying an l-level DWT to the discrete baseband signal s(t), which represents the spread-spectrum signal corresponding to one complete navigation symbol period. This choice allows us to embed the watermark into the fine structure of the symbol itself, prior to modulation onto the carrier. This yields a set of detail coefficient sub-bands $\left\{D_{1},D_{2},\ldots ,D_{l}\right\}$ and a final approximation coefficient sub-band $A_{l}$.

For the implementation of WAWA, the Daubechies (db) wavelet family is selected as the basis function. This choice is motivated by several key properties that are highly advantageous for this application: orthogonality, which is a prerequisite for the energy-based embedding model; compact support, which helps in localizing the analysis in time; and regularity, which contributes to a less perceptible watermark. The choice of the specific wavelet (e.g., db4) and the decomposition level are fixed system parameters known to all compliant receivers. WAWA selects one or more of these sub-bands, denoted $D_{target}$, for watermark embedding. Typically, sub-bands with lower original signal energy are chosen to maximize the watermark’s robustness.

To control the watermark’s power and its impact on the original signal, a power allocation factor α is defined as the ratio of the watermark power $P_{w}$ to the power of the original signal *C*,

(2) $$\begin{array}{c} \alpha =P_{w}/C \end{array}$$

The normalized watermark sequence w(t) is then scaled and added to the target sub-band coefficients $D_{target}\left(t\right)$ to produce the modified coefficients $D_{target}^{\prime }\left(t\right)$,

(3) $$\begin{array}{c} D_{target}^{\prime }\left(t\right)=D_{target}\left(t\right)+\sqrt{\alpha C}\;\cdot \;w\left(t\right) \end{array}$$

It is crucial to note that this additive operation is performed in the wavelet coefficient domain. Both $D_{target}\left(t\right)$ and the scaled watermark w(t) represent sequences of coefficients at this stage, not time-domain signals. This ensures the watermark energy is precisely injected into the desired spectral sub-band. This additive embedding strategy also relies on the orthogonality of the chosen wavelet basis, which ensures that the energy of the watermark adds linearly to the energy of the original signal, allowing the correlation loss to be precisely controlled by the parameter α according to Parseval’s theorem.

Finally, the modified coefficients $D_{target}^{\prime }\left(t\right)$ and all other unmodified coefficients ($A_{l}$, $D_{other}$) are used to reconstruct the final watermarked signal $s_{w}\left(t\right)$ via the Inverse Discrete Wavelet Transform (IDWT),

(4) $$\begin{array}{c} s_{w}\left(t\right)=\mathrm{IDWT}\left(A_{l},D_{target}^{\prime },D_{other}\right) \end{array}$$

#### 2.2.3. Authentication Watermark Sequence Design

The watermark sequence w(t) is designed to carry the authentication information robustly and efficiently. It employs a composite structure that combines the raw authentication bits with spread spectrum modulation. The generation process is shown in the gray-highlighted portion of Figure 8.

First, the authentication payload is defined. The sender signs a segment of the navigation message, $M_{seg}$, concatenated with a timestamp *T*, using the private key $K_{priv}$ to generate the digital signature Sig,

(5) $$\begin{array}{c} Sig=\mathrm{Sign}(K_{priv},M_{seg}||T) \end{array}$$

This signature, along with any necessary overhead (e.g., metadata), forms the complete authentication sequence $w_{au}\left(k\right)$, with a total length of $N_{au}$ bits.

Next, this bit sequence is mapped to the time domain. We define a key parameter, $N_{bpsym}$ (bits per symbol), as the average number of authentication bits embedded within one navigation symbol period $T_{sym}$. The duration of a single authentication bit, $T_{bit}$, is therefore

(6) $$\begin{array}{c} T_{bit}=\frac{T_{sym}}{N_{bpsym}} \end{array}$$

Finally, each authentication bit $w_{au}\left(k\right)$ is spread using a public and fixed spreading sequence $C_{w}\left(t\right)$ to enhance its robustness for subsequent embedding and extraction. The length of this spreading sequence, $L_{cw}$, depends on the original signal’s chipping rate $R_{c}$, the number of DWT levels *l*, and the bit duration $T_{bit}$,

(7) $$\begin{array}{c} L_{cw}=\frac{R_{c}}{2^{l}}T_{bit} \end{array}$$

The final watermark sequence w(t) is generated by modulating each bit with the spreading sequence

(8) $$\begin{array}{c} w\left(t\right)=\sum_{k}w_{au}\left(k\right)\;\cdot \;C_{w}\left(t-kT_{bit}\right) \end{array}$$

By using a public, fixed spreading code $C_{w}\left(t\right)$, the receiver can perform despreading with minimal complexity and memory, as it only needs to store this known sequence.

#### 2.2.4. Receiver Watermark Extraction and Dual-Path Verification

**Figure 9:**
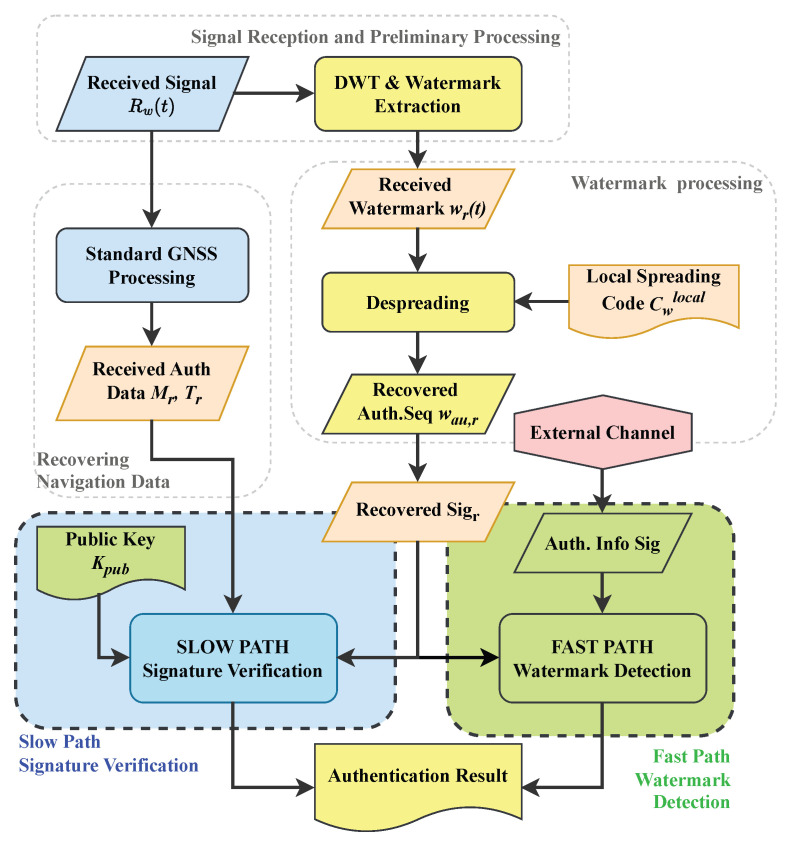
Receiver flowchart showing watermark extraction and the dual-path authentication process.

The receiver’s main task is to extract the embedded signature from the received signal and perform verification. The dual-path framework offers flexibility to trade authentication latency for reliance on external communication channels. The receiver processing flow is detailed in Figure 9.

After acquiring and tracking the GNSS signal, the receiver performs an *l*-level DWT on the baseband signal $r_{w}\left(t\right)$ to obtain the target sub-band coefficients $D_{target}^{received}$. Using a local replica of the public spreading code $C_{w}^{local}\left(t\right)$, it then performs a correlation-based despreading operation to obtain a soft-decision value $z_{bit}\left(k\right)$ for each embedded authentication bit,

(9) $$\begin{array}{c} z_{bit}\left(k\right)=\sum_{j=1}^{L_{cw}}D_{target}^{received}\left(k,j\right)\;\cdot \;C_{w}^{local}\left(j\right) \end{array}$$

where $D_{target}^{received}\left(k,j\right)$ is the *j*-th coefficient in the target sub-band corresponding to the *k*-th bit interval. Since $C_{w}$ is public and fixed, the receiver only needs to buffer the current soft-decision values, leading to minimal memory usage. From here, verification can follow one of two paths.

(1) Slow Path Verification: Standalone Signature Check

This path is designed for standalone receivers without access to external channels. The verification process begins by making a hard decision on each soft value $z_{bit}\left(k\right)$ to recover the estimated authentication bits ${\hat{w}}_{au}\left(k\right)$, for example, based on the sign

(10) $$\begin{array}{c} {\hat{w}}_{au}\left(k\right)=\mathrm{sign}\left(z_{bit}\left(k\right)\right) \end{array}$$

The receiver accumulates these recovered bits over time to reconstruct the complete estimated signature, $\hat{Sig}$. Once the corresponding navigation message segment $M_{seg}$ and timestamp *T* are also received, the receiver can immediately perform the standard digital signature verification using the pre-stored public key $K_{pub}$,

(11) $$\begin{array}{c} Result=\mathrm{Verify}(K_{pub},M_{seg}||T,\hat{Sig}) \end{array}$$

A true result from the ‘Verify’ function confirms the authenticity and integrity of the navigation message. The key advantage here is immediacy: unlike TESLA, this verification does not require waiting for a future key disclosure.

(2) Fast Path Verification: Assisted Correlation Detection

This path is designed for network-assisted receivers that can obtain the authentic signature Sig in advance via a fast external channel (e.g., a cellular network). This allows for a more rapid and robust authentication process.

With the authentic signature Sig, the receiver constructs a local, error-free reference bit sequence $w_{au}^{local}\left(k\right)$. It then correlates this reference sequence with the stream of real-time soft-decision values $z_{bit}\left(k\right)$ to compute a final detection statistic, $Z_{seq}$,

(12) $$\begin{array}{c} Z_{seq}=\sum_{k=1}^{N_{au}}z_{bit}\left(k\right)\;\cdot \;w_{au}^{local}\left(k\right) \end{array}$$

This approach provides significant processing gain by correlating over the entire signature sequence, which greatly improves the detection Signal-to-Noise Ratio (SNR) compared to the bit-by-bit hard decisions of the slow path. Consequently, high authentication reliability can be achieved even at lower Carrier-to-Noise density ratios ($C/N_{0}$). The authentication speed is limited only by the latency of the external channel, enabling near real-time verification.

## 3. Results

This section presents a theoretical performance analysis of the proposed WAWA scheme. The analysis uses parameters consistent with the Galileo E1-B signal as a case study to evaluate the core performance metrics: watermark detection reliability, effective channel bandwidth, and the impact of the watermark on the primary navigation signal.

### 3.1. Watermark Detection Performance

The reliability of detecting the embedded watermark is crucial for both verification paths. We analyze the performance of each path separately.

#### 3.1.1. Slow Path: Bit Error Rate (BER) Analysis

The performance of the slow path depends on the ability to correctly decode individual authentication bits, which is quantified by the Bit Error Rate (BER). In an Additive White Gaussian Noise (AWGN) channel, the BER is a function of the Signal-to-Noise Ratio (SNR) per bit, $SNR_{bit}$,

(13) $$\begin{array}{c} SNR_{bit}=\frac{E_{bit}}{N_{0}}=\frac{\alpha CT_{bit}}{N_{0}}=\frac{\alpha C}{N_{0}}\;\cdot \;\frac{T_{sym}}{N_{bpsym}} \end{array}$$

where $E_{bit}$ is the energy per watermark bit, and $N_{0}$ is the single-sided noise power spectral density.

Assuming the watermark bits are modulated using Binary Phase Shift Keying (BPSK), the theoretical BER for the WAWA slow path is given by the standard Q-function

(14) $$\begin{array}{c} BER_{WAWA}=Q\left(\sqrt{2\;\cdot \;SNR_{bit}}\right)=Q\left(\sqrt{2\frac{\alpha }{N_{bpsym}}\frac{C}{N_{0}}T_{sym}}\right) \end{array}$$

The probability of successfully authenticating an entire signature of $N_{au}$ bits without error is

(15) $$\begin{array}{c} P_{d\_slow}\approx {\left(1-BER_{WAWA}\right)}^{N_{au}} \end{array}$$

#### 3.1.2. Fast Path: Correlation Detection Performance

The fast path correlates the received signal with a known authentic signature. Its performance is characterized by the detection probability ($P_{d}$) for a given false alarm probability ($P_{fa}$), which depends on the accumulated SNR over the entire sequence, $SNR_{seq}$,

(16) $$\begin{array}{cc} SNR_{seq} & =\frac{{\left(E\left[Z_{seq}|\mathrm{signal}\right]\right)}^{2}}{\mathrm{Var}(Z_{seq}|\mathrm{signal})}\approx \frac{{\left(N_{au}\sqrt{E_{bit}}\right)}^{2}}{N_{au}N_{0}/2}\\ \; & =2\alpha \frac{C}{N_{0}}T_{au} \end{array}$$

where $T_{au}=N_{au}T_{bit}$ is the total time to transmit the signature. The detection probability is given by

(17) $$\begin{array}{c} P_{d\_fast}=Q\left(Q^{-1}\left(P_{fa}\right)-\sqrt{SNR_{seq}}\right) \end{array}$$

where $Q^{-1}\left(\;\cdot \;\right)$ is the inverse Q-function.

#### 3.1.3. Performance Evaluation

For evaluation, we compare WAWA’s performance with the uncoded data bit BER of Galileo OSNMA. Neglecting channel coding, the BER for OSNMA data bits [15] is given by

(18) $$\begin{array}{c} BER_{Data}=Q\left(\sqrt{2\frac{C}{N_{0}}T_{sym}}\right) \end{array}$$

We use parameters based on the Galileo E1-B signal and a representative cryptographic configuration, as summarized in Table 1. The signature length of 672 bits is based on the design in TrustCNAV [25], which provides a concrete example of a modern certificateless PKC scheme suitable for GNSS.

**Figure 10:**
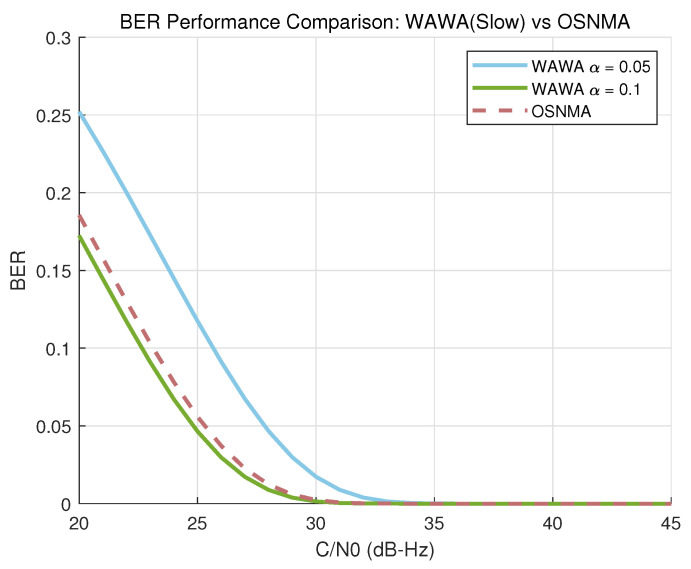
Bit Error Rate (BER) performance of the WAWA slow path versus uncoded OSNMA data bits as a function of $C/N_{0}$.

**Figure 11:**
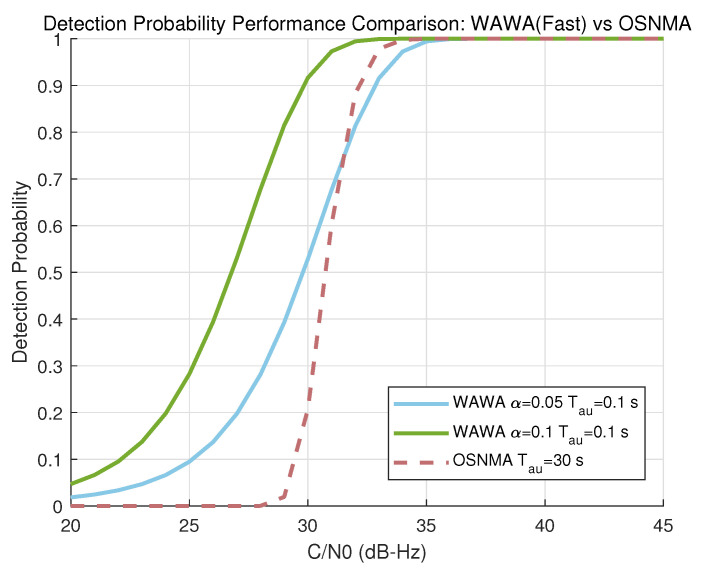
Detection probability ($P_{d}$) of the WAWA fast path (for $P_{fa}=10^{-6},T_{au}=1\;s$) versus uncoded OSNMA BER as a function of $C/N_{0}$.

Figure 10 plots the BER of the WAWA slow path against the uncoded OSNMA BER for two different power allocation factors (α=0.05 and α=0.1) and an embedding rate of $N_{bpsym}=1$. Figure 11 shows the detection probability of the WAWA fast path for a false alarm rate of $P_{fa}=10^{-6}$ and a rapid authentication interval of $T_{au}=1$ s.

The results show that the WAWA slow path’s BER is dependent on the power allocation factor α. At α=0.1, its performance is comparable to that of uncoded OSNMA bits. The fast path exhibits highly reliable performance, achieving near-certain detection ($P_{d}\approx 1$) even at moderate $C/N_{0}$ values over a short 1-s interval.

### 3.2. Effective Channel Bandwidth

**Figure 12:**
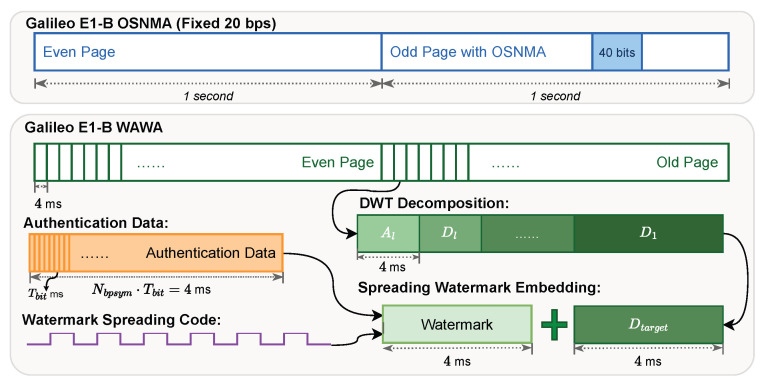
Conceptual comparison of the data embedding structures for OSNMA and WAWA within the Galileo E1-B signal timing framework.

A primary innovation of WAWA is the establishment of a high-bandwidth authentication channel. OSNMA is limited to a fixed data rate, embedding 40 bits of authentication data every 2 s, which results in an effective bandwidth of 20 bps [15]. Figure 12 provides a conceptual illustration of this structural difference.

In contrast, the effective bandwidth of the WAWA watermark channel, $R_{wawa}$, is determined by the number of bits embedded per symbol, $N_{bpsym}$,

(19) $$\begin{array}{c} R_{wawa}=\frac{N_{bpsym}}{T_{sym}} \end{array}$$

**Figure 13:**
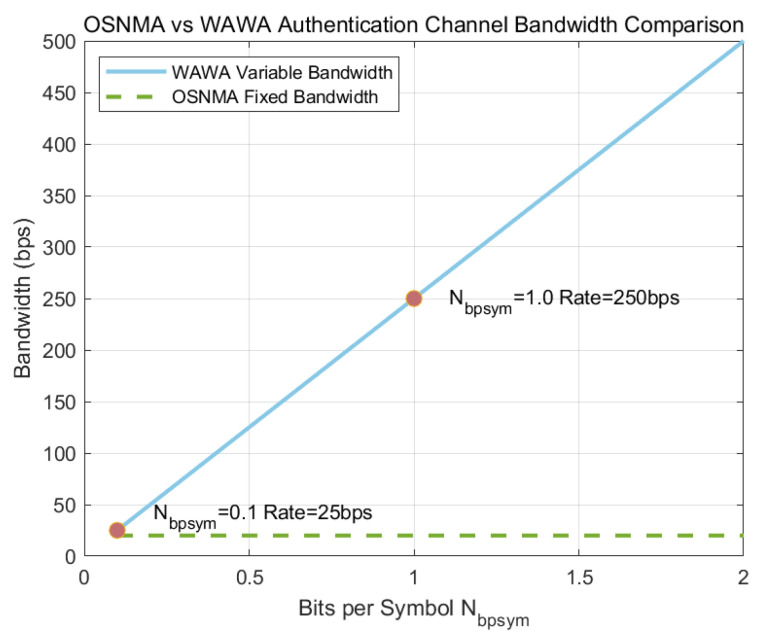
Effective watermark bandwidth comparison for the Galileo E1-B signal: WAWA vs. $N_{bpsym}$ compared to the fixed rate of OSNMA.

Figure 13 plots this relationship for the Galileo E1-B signal ($T_{sym}=4$ ms). The results show that WAWA’s bandwidth scales linearly with $N_{bpsym}$ and can significantly surpass the fixed, low rate of OSNMA. For instance, embedding just one bit per symbol ($N_{bpsym}=1$) yields a bandwidth of 250 bps, over an order of magnitude greater than OSNMA.

### 3.3. Impact on Legacy GNSS Receivers

A foundational requirement for any GNSS signal component is a thorough understanding of its impact on existing, legacy receivers. Backward compatibility is paramount for the technology’s adoption. To provide a comprehensive evaluation of this critical aspect for WAWA, this section presents a quantitative investigation into its effects. We first establish and validate the theoretical model for the watermark’s primary physical effect—correlation loss—and then analyze the downstream consequences of this loss on the receiver’s code tracking precision.

#### 3.3.1. Correlation Loss: Theory and Validation

For a legacy receiver that is unaware of the WAWA scheme, the embedded watermark effectively acts as a source of structured, low-power noise that is uncorrelated with the local PRN code replica. This inevitably leads to a reduction in the correlation peak power, an effect quantified as Correlation Loss (CL). Our theoretical model for this loss is a direct function of the authentication energy ratio, α:

(20) $$\begin{array}{c} {\mathrm{CL}}_{\mathrm{WAWA}}=10\log_{10}\left(1-\alpha \right)\;\left(\mathrm{dB}\right) \end{array}$$

**Figure 14:**
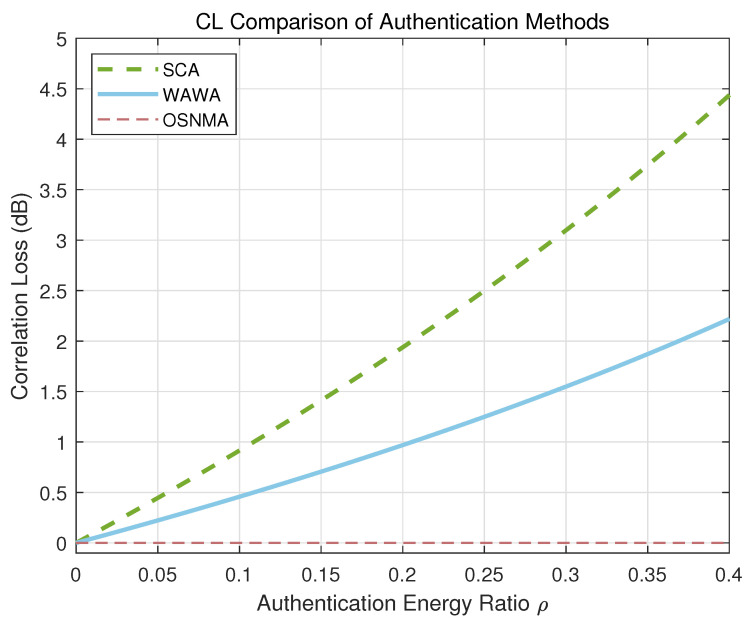
Correlation loss as a function of the Authentication Energy Ratio (ρ) for WAWA and SCA schemes. OSMA has zero correlation loss.

This model highlights a key design advantage of WAWA. As shown in Figure 14, for an equivalent portion of signal energy allocated to authentication, the resulting correlation loss is inherently lower than that of a Chimera-like SCA scheme, which modifies the signal in the time domain.

**Figure 15:**
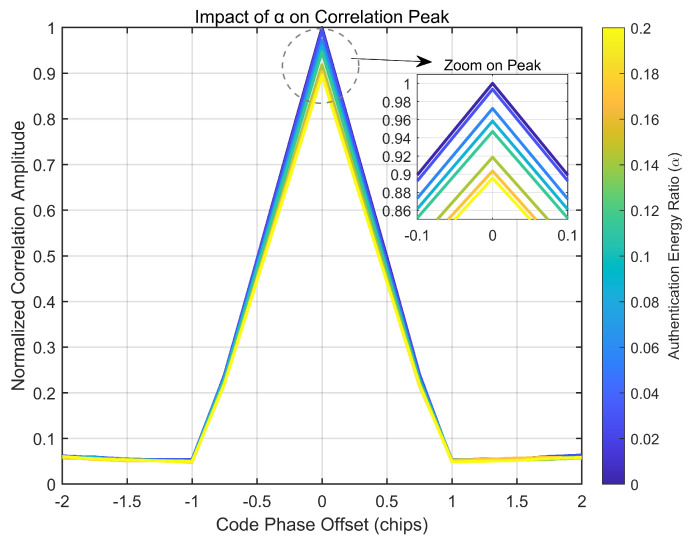
Impact of the authentication energy ratio (α) on the normalized correlation peak. The inset provides a zoomed-in view, clearly showing the peak amplitude reduction as more energy is allocated to the watermark.

To confirm the accuracy of this theoretical model, we performed a series of baseband-level simulations. First, a qualitative view of the effect is provided in Figure 15, which visually demonstrates how the normalized correlation peak amplitude attenuates as more energy is allocated to the watermark via an increasing α.

**Figure 16:**
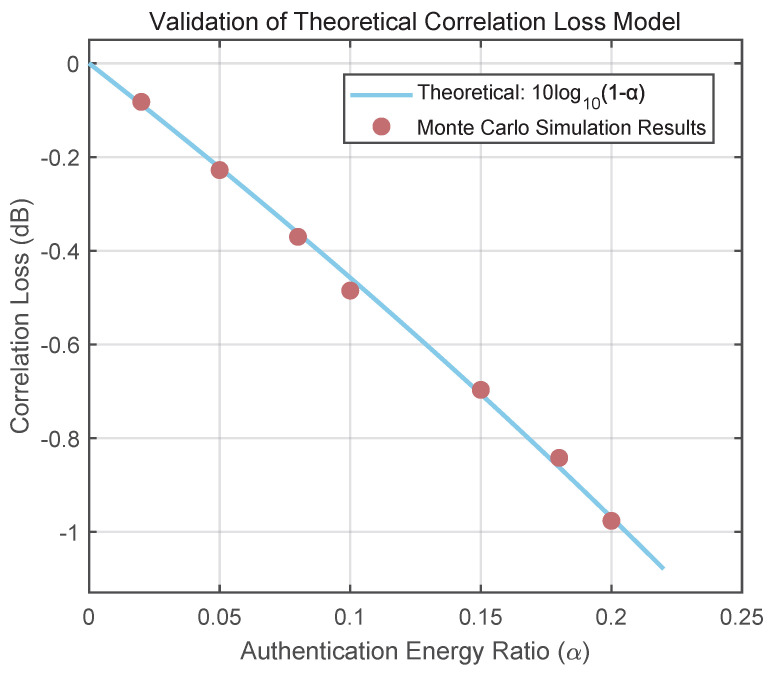
Validation of the theoretical correlation loss model. The red dots represent Monte Carlo simulation results, which align closely with the theoretical prediction (solid blue line).

Next, to achieve a rigorous quantitative validation, a Monte Carlo simulation was performed to test the precise accuracy of Equation (20). The results, presented in Figure 16, reveal an excellent agreement between the simulated data points and the theoretical curve. This validation is crucial, as it confirms that α is not merely a theoretical concept but a reliable and predictable engineering parameter for controlling the scheme’s impact.

#### 3.3.2. Impact on Code Tracking Precision

With the correlation loss model now rigorously validated, we can quantitatively project its operational consequences for a legacy receiver. The most critical of these is the degradation of code tracking precision in the Delay-Locked Loop (DLL), as this directly affects the accuracy of pseudorange measurements. The DLL’s performance is fundamentally governed by the effective Carrier-to-Noise density ratio, ${\left(C/N_{0}\right)}_{\mathrm{eff}}$, which is directly reduced by the correlation loss

(21) $$\begin{array}{c} {\left(C/N_{0}\right)}_{\mathrm{eff}}=\left(C/N_{0}\right)\;\cdot \;\left(1-\alpha \right) \end{array}$$

The relationship between this effective $C/N_{0}$ and the resulting tracking error is captured in the standard model for DLL thermal noise jitter. The standard deviation of the error, $\sigma_{\mathrm{DLL}}$, can be approximated as

(22) $$\begin{array}{c} \sigma_{\mathrm{DLL}}\approx c\;\cdot \;\sqrt{B_{L}d\frac{1}{2{\left(C/N_{0}\right)}_{\mathrm{eff}}}\left(1+\frac{1}{T_{int}{\left(C/N_{0}\right)}_{\mathrm{eff}}}\right)}\;\left(\mathrm{meters}\right) \end{array}$$

where *c* is the speed of light, $B_{L}$ is the loop noise bandwidth, *d* is the early-late correlator spacing (in chips), and $T_{int}$ is the coherent integration time.

**Figure 17:**
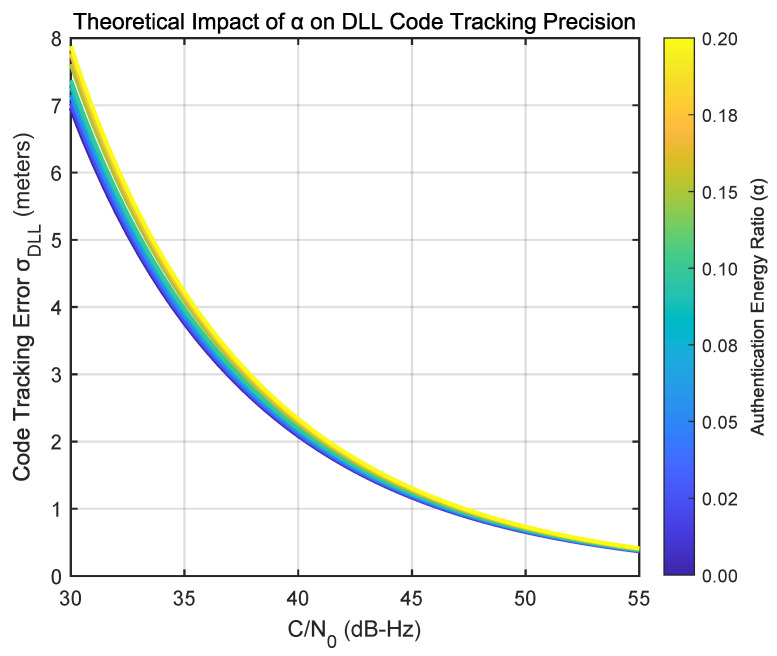
Theoretical impact of the authentication energy ratio (α) on DLL code tracking precision. The plot shows the tracking error standard deviation ($\sigma_{\mathrm{DLL}}$) as a function of $C/N_{0}$ for various α values.

By substituting our validated expression for ${\left(C/N_{0}\right)}_{\mathrm{eff}}$ into this formula, we can generate a precise theoretical prediction of the impact of α on pseudorange precision. Figure 17 plots this relationship for a set of typical receiver parameters ($B_{L}=1$ Hz, d=0.5 chips, $T_{int}=4$ ms). The resulting performance curves illustrate a clear and manageable trade-off. For instance, at a typical $C/N_{0}$ of 45 dB-Hz, even a relatively high energy allocation of α=0.1 (a −0.46 dB correlation loss) results in a precision degradation of only a few decimeters. This analysis provides a robust framework for system designers to make informed decisions, balancing the required level of authentication robustness against the acceptable impact on legacy receiver performance.

## 4. Discussion

The results presented in the previous section demonstrate that the WAWA scheme offers a unique set of capabilities that directly address the primary limitations of existing GNSS authentication methods. This section interprets these findings in the broader context of GNSS security, comparing WAWA’s performance trade-offs against the established NMA and SCA paradigms.

### 4.1. Immediacy of Verification and Its Implications

The most significant advantage of WAWA is its ability to provide immediate authentication. As shown in Figure 10 and Figure 11, this immediacy is achieved without compromising performance under typical operating conditions. The slow path can achieve a BER comparable to the uncoded bits of OSNMA, while the fast path offers exceptionally high detection reliability. This capability directly resolves the chief operational drawback of TESLA-based NMA schemes: the inherent verification latency of tens of seconds.

This elimination of delay has profound implications for time-critical applications. For autonomous vehicles, drones, or critical infrastructure timing, a 30-second window of uncertainty is a significant security risk. An attacker could inject false information during this period, causing immediate and potentially catastrophic effects before the data can be invalidated. WAWA closes this window of vulnerability. The dual-path framework further enhances this flexibility. The slow path guarantees a baseline autonomous capability for any receiver, while the fast path provides a near real-time, high-robustness solution for connected systems, where low latency is the highest priority.

### 4.2. Bandwidth as a Key Enabler for Stronger Cryptography

The bandwidth analysis in Figure 13 highlights the fundamental enabler of WAWA’s architecture: the creation of a high-throughput physical layer channel. This high bandwidth is what makes it feasible to transmit a complete public-key signature in a timely manner—a task that is simply impossible with OSNMA’s fixed 20 bps data rate. By moving authentication from the slow navigation message bits to the much faster physical layer symbols, WAWA unlocks the ability to use more robust cryptographic primitives without incurring unacceptable latency.

Furthermore, the tunable parameter $N_{bpsym}$ introduces a valuable degree of freedom for system designers. As shown by the relationship in Equation 13, a designer can choose to decrease $N_{bpsym}$ (lowering the bandwidth) to significantly boost the robustness ($SNR_{bit}$) of the slow path for receivers in weak signal environments. This adaptability allows the system to be optimized for different use cases and channel conditions, a flexibility not available in fixed-rate schemes like OSNMA.

### 4.3. Resource Efficiency: Overcoming the Memory Barrier

Beyond its temporal advantages, WAWA’s most significant practical contribution is its profound resource efficiency, a distinction highlighted in Table 2.

This stands in stark contrast to SCA schemes like Chimera. WAWA’s symbol-level, real-time processing architecture fundamentally obviates the need for the megabyte-scale memory buffers essential for SCA’s delayed verification. This high memory and power overhead has historically been a prohibitive barrier for integrating strong cryptographic authentication into the vast market of resource-constrained devices, such as IoT sensors. WAWA dismantles this barrier, making high-security authentication practical for a much broader class of receivers.

These advantages, however, are balanced by a clear set of design trade-offs. Unlike the non-intrusive NMA approach, WAWA introduces a controlled correlation loss for legacy receivers. Yet, as quantified in Figure 14 and validated in Section 3.3, this impact is not only tunable via the power allocation parameter α but is also demonstrably less severe than that of SCA for an equivalent authentication energy. This provides system designers with a transparent, predictable mechanism to balance authentication robustness against backward compatibility. Furthermore, while the DWT and PKC operations introduce a moderate computational overhead compared to NMA, this is a modest demand for modern embedded processors. It represents a justifiable engineering trade-off for the immense security gains of eliminating verification latency and time-synchronization vulnerabilities.

The choice of key system parameters involves a multi-dimensional trade-off. For instance, increasing the DWT decomposition level allows for finer spectral placement of the watermark but increases computational complexity. Increasing $N_{bpsym}$ boosts bandwidth but reduces the per-bit energy for the slow path (as shown in Equation (13)), requiring a higher $C/N_{0}$ or α. The optimal parameter set depends on the target application scenario. For example, a UAV operating in an open-sky environment, which prioritizes high bandwidth, would favor a larger value for $N_{bpsym}$.Conversely, a receiver in an urban canyon, requiring maximum robustness against weak signal conditions, would benefit from decreasing $N_{bpsym}$ while simultaneously increasing α to boost the per-bit SNR.

### 4.4. Security Considerations, Limitations, and Future Work

From a security perspective, WAWA’s reliance on public-key digital signatures provides strong protection against forgery, as creating a valid signature without the private key is computationally infeasible under standard cryptographic assumptions. An adversary aware of the scheme might attempt a watermark-aware attack, such as selective removal of the targeted DWT sub-band. However, this is challenging without causing significant distortion to the legacy signal components. Timed replay attacks are fundamentally mitigated because WAWA’s immediate verification closes the window of vulnerability exploited in TESLA-based systems. The management of public keys ($K_{pub}$) is a critical component of the system’s root of trust. WAWA’s architecture is compatible with established Public Key Infrastructure (PKI) models, such as that proposed in TrustCNAV, where keys are managed through a trusted out-of-band channel or periodic broadcasts. For the fast path, the trusted external channel must be independently secured (e.g., via TLS/SSL) to prevent Man-in-the-Middle (MITM) attacks. Furthermore, looking toward future threats, the rise in quantum computing poses a long-term risk to classical cryptography. While a full transition to Post-Quantum Cryptography (PQC) is beyond the scope of this paper, it is noteworthy that the high-bandwidth channel created by WAWA is inherently more adaptable to the larger signature sizes typical of PQC schemes. This flexibility represents a significant advantage for the long-term viability of the architecture.

Despite its advantages, it is important to acknowledge the scheme’s limitations. The primary trade-off is the introduction of a controllable correlation loss, which, as analyzed in Section 3.3, affects the performance of legacy receivers. Our analysis was also primarily conducted under AWGN channel conditions. The performance of WAWA in complex multipath and fading environments, while hypothesized to degrade gracefully along with the host signal, requires further investigation and is a critical area for future research. Finally, the practical deployment of WAWA would necessitate modifications to the GNSS space and ground segments for watermark embedding and synchronization. This represents a significant long-term challenge that requires coordination with international standardization bodies, positioning WAWA as a next-generation authentication concept rather than an immediate overlay system.

## 5. Conclusions

This paper confronted the defining limitations of the two dominant paradigms in GNSS signal authentication. Navigation Message Authentication (NMA), epitomized by TESLA-based protocols, is fundamentally hampered by high verification latency and a critical security vulnerability tied to time synchronization. Conversely, Spreading Code Authentication (SCA) schemes impose prohibitive memory buffering requirements on receivers, restricting their use in the vast and growing market of low-cost, resource-constrained devices.

To resolve this impasse, we have proposed, designed, and analyzed WAWA, a novel Wavelet Analysis-based Watermarking Authentication architecture. WAWA’s core innovation is its strategic use of the wavelet domain to create a high-capacity channel within the physical signal layer, enabling the transmission of a complete public-key signature. This design systematically dismantles the shortcomings of previous approaches. It achieves immediate, symbol-level verification, which completely eliminates the latency and time-synchronization risks of TESLA. Simultaneously, its real-time, symbol-by-symbol processing obviates the need for the large memory buffers that have hindered the practicality of SCA.

Our performance analysis confirms that WAWA provides robust authentication performance while giving system designers direct control over the trade-off between security and backward compatibility via a single power allocation parameter. The dual-path verification framework further enhances its flexibility, offering a high-performance solution for network-assisted receivers while maintaining full capability for standalone devices.

In comparison to existing schemes, WAWA establishes a new and highly practical paradigm for GNSS authentication. It decisively resolves the critical issues of immediacy, security, and resource efficiency that have long constrained the field. By uniquely combining these attributes, WAWA provides a robust and forward-looking framework for guaranteeing the integrity of PNT sensor data. It thereby enables a new class of secure, distributed applications, from city-scale IoT deployments to fully autonomous systems that depend on trustworthy authentication performed efficiently at the network edge. 

## Figures and Tables

**Table 1:** Key parameters used in the performance evaluation.

Parameter	Symbol	Value
Symbol Duration	$T_{sym}$	4 ms
Signature Length	$N_{au}$	672 bits
Bits per Symbol	$N_{bpsym}$	1
False Alarm Probability (Fast Path)	$P_{fa}$	$10^{-6}$
Authentication Interval (Fast Path)	$T_{au}$	1 s

**Table 2:** Qualitative comparison of GNSS authentication schemes: NMA (OSNMA), SCA (Chimera), and the proposed WAWA scheme.

Feature	OSNMA (NMA Example)	Chimera (SCA Example)	WAWA (This Work)
Authentication Level	Navigation Message	Spreading Code/Physical Layer	Navigation Message via Physical Layer
Typical Verification Latency	High ( 30 s)	High (waits for NMA key)	Immediate (Slow/Fast Path)
Receiver Memory Req.	Low	Very High (MBs)	Low
Receiver Comp. Complexity	Low	Moderate	Moderate (DWT)
Correlation Loss	None	High: $20\log_{10}\left(1-DF\right)$ dB	Moderate: $10\log_{10}\left(1-\alpha \right)$ dB
Time Sync. Dependency	High (Vulnerable)	High (via NMA)	None
Primary Advantages	Backward compatibility	Chip-level protection	Immediacy, Low Memory, No Sync. Risk
Primary Disadvantages	Latency, Sync. Risk, Low BW	High Memory, High Corr. Loss	Introduces Corr. Loss, PKI Management, Space Segment Mod.

## Data Availability

The data presented in this study are available in the article. The study did not generate new publicly achievable datasets.

## Abbreviations

The following abbreviations are used in this manuscript:

BER	Bit Error Rate
DLL	Delay-Locked Loop
DWT	Discrete Wavelet Transform
GNSS	Global Navigation Satellite System
IDWT	Inverse Discrete Wavelet Transform
NMA	Navigation Message Authentication
OSNMA	Open Service Navigation Message Authentication
SCA	Spreading Code Authentication
SNR	Signal-to-Noise Ratio
TESLA	Timed Efficient Stream Loss-tolerant Authentication
PNT	Positioning, Navigation, and Timing
PKC	Public Key Cryptography
PKI	Public Key Infrastructure
WAWA	Wavelet Analysis based Watermarking Authentication
